# Complete mitogenome of *Gobiopterus lacustris* (Perciformes: Gobiidae)

**DOI:** 10.1080/23802359.2018.1536456

**Published:** 2018-11-26

**Authors:** Chengqin Huang, Zhongdian Dong, Shuisheng Long, Shunkai Huang, Hairui Zhang, Chun Wang, Yusong Guo, Zhongduo Wang

**Affiliations:** Key Laboratory of Aquaculture in South China Sea for Aquatic Economic Animal of Guangdong higher Education Institutes, Fisheries College, Guangdong Ocean University, Zhanjiang, China

**Keywords:** *Gobiopterus lacustris*, mitogenome, phylogenetics

## Abstract

In this study, combining the gonad transcripts of the both sexes by RNA-Seq with the DNA sequences by conventional PCRs, we have determined the complete mitogenome of *Gobiopterus lacustris* collected from the mangrove waters of the Leizhou Peninsula in tropical South China. This mitochondrial genome is 16593 bp in length and consists of 37 genes in the typical vertebrate mitochondrial gene arrangement. This study will contribute on the phylogenetics of genus *Gobiopterus* and the other genera of Gobiidae.

*Gobiopterus lacustris* (Perciformes: Gobiidae) is a small transparent species, mainly distributed in the freshwater lake of Luzon in Philippines (Aquino et al. 2011) and the mangroves areas of Leizhou Peninsula of China (Liao et al. 2016). Based on the genetic differentiation analysis of *COI* gene and D-loop region, a significant differentiation occurred of *G. lacustris* between eastern and western Leizhou Peninsula (Wang et al. 2017). As far as we know, no mitochondrial genome sequences belonging to genus *Gobiopterus* is currently listed in GenBank database. In this paper, the complete mitogenome sequence of *G. lacustris* (GenBank accession no. MH512902) was reported at the first time.

The *G. lacustris* specimen used in this study was collected from National Mangrove Nature Reserve in Leizhou Peninsula, Guangdong Province, China (21°34′11″N, 109°45′24″E). The muscle was used to extract DNA (Guo et al. 2016), while the total RNA was isolated from the gonad using the TRIzol reagent (Invitrogen, Carlsbad, CA) and used for transcriptomic sequencing (RNA-Seq) (Wang et al. 2009). The typical specimen and DNA were deposited in our laboratory. According to the sequences of RNA-Seq, five pairs of primers for conventional PCR were designed. The amplified DNA products were sequenced to fill the gaps and substitute the 3′ transcript ends between the 16S rRNA and *ND1* gene, *ND2* and *COX1*gene, *COX1* and *COX2* gene, *ATP6* and *COX3* gene, Cytb gene and the control region, the control region and the 12s rRNA gene.

As a result, we have determined the mitogenome of *Gobiopterus lacustris*. The circle genome is 16 593 bp in length, with the base composition of 27.4% A, 27.1% T, 16.6% G, and 28.9% C. It comprises 13 typical vertebrate protein-coding genes, 22 transfer RNA genes, 2 ribosomal RNA genes, and 1 control region. The mitochondrial gene arrangement of *G. lacustris* was identical to other gobies (Kim et al. 2004a,b, Liu et al. 2012). Except for eight tRNA and NADH dehydrogenase subunit 6 (ND6), all other mitochondrial genes were encoded on the heavy strand. All protein genes had an ATG start codon except for the cytochrome oxidase subunit I gene with GTG as an initiation codon. five complete stop codons (TAG, TAA, AGA, TCT, and AGG) and three incomplete stop codons (T–) were used in the protein-coding genes. All tRNA genes can be folded into the standard cloverleaf secondary structures except for tRNA^Ser^.

Phylogenetic analysis based on the mitochondrial genomes of 13 species by MEGA6 showed that *G. lacustris* clustered with the clade of *T. bifasciatus* supported by a high bootstrap value (99%) and the family Gobiidae was not a monophyly (Figure 1), in which the similar mtDNA sequences of the order Perciformes were downloaded from GenBank through BLASTN and zebrafish (*Danio rerio*) of the order Cypriniformes was used as outgroup.

**Figure 1.**
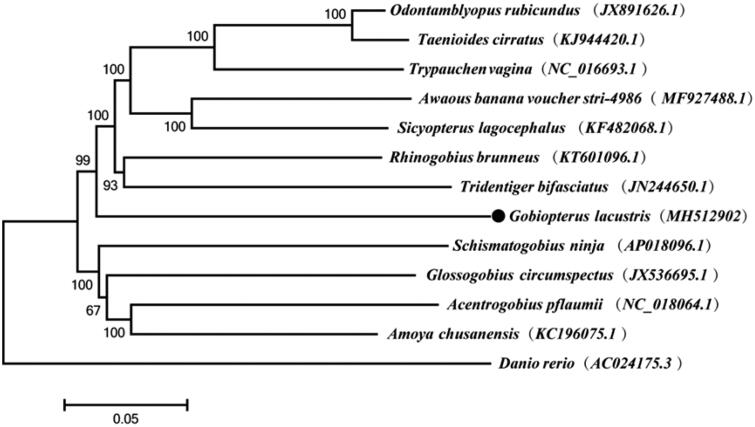
Neighbor-joining phylogenetic tree based on mitochondrial genome sequences. All the bootstrap values after 1000 iteration are indicated at the nodes.
